# Effects of a complex intervention on agitation and aggression in people living with dementia and mild cognitive impairment in shared-housing arrangements: results for a secondary outcome of the multicenter, cluster-randomized controlled DemWG study

**DOI:** 10.1186/s12888-026-07810-x

**Published:** 2026-01-24

**Authors:** Carolin Donath, André Kratzer, Elmar Graessel, Antonia Keck, Serhat Günay, Janissa Altona, Julia Misonow, Susanne Stiefler, Annika Schmidt, Karin Wolf-Ostermann

**Affiliations:** 1 Center for Health Services Research in Medicine, Department of Psychiatry and Psychotherapy, Uniklinikum Erlangen, Friedrich-Alexander-Universität Erlangen-Nürnberg (FAU), Erlangen, Germany; 2 Competence Center for Clinical Trials Bremen (KKSB), University of Bremen, Bremen, Germany; 3 Institute for Public Health and Nursing Science (IPP), University of Bremen, Bremen, Germany; 4 Health Sciences Bremen, Bremen, Germany

**Keywords:** Dementia, Mild cognitive impairment, Psychosocial intervention, Complex intervention, Behavioral and psychological symptoms of dementia, BPSD, Quality of life, Shared-housing arrangements, RCT

## Abstract

**Background:**

Although some studies have suggested that homelike care environments can have beneficial effects on people living with dementia (PlwDs) and people living with mild cognitive impairment (PlwMCIs), studies on effects of a non-pharmacological, psychosocial intervention on behavioral and psychological symptoms of dementia (BPSDs) in the setting of shared-housing arrangements (SHAs) are still lacking.

**Methods:**

In the prospective, multicenter, mixed-methods, cluster-randomized controlled DemWG study, 97 SHAs comprising 341 PlwDs or PlwMCIs were randomly assigned to either the intervention group (IG) or control group (CG). The complex intervention consisted of the education of nursing staff in SHAs (Component A), the education of general practitioners (Component B), and the multicomponent, psychosocial group intervention MAKS-mk+ (Component C). BPSDs (secondary outcome of the DemWG study) were assessed with the Cohen-Mansfield Agitation Inventory-Short Form (CMAI-SF) and the Neuropsychiatric Inventory-Nursing Home Version (NPI-NH) at baseline (t0), 6 months after baseline (t1) and 12 months after baseline (t2). Unadjusted and adjusted generalized estimating equations (GEE) models were computed to investigate possible effects of the complex intervention on the outcome variables at t1 or t2.

**Results:**

In the intention-to-treat (ITT) sample, the adjusted GEE models showed that participants in the IG had a significantly lower CMAI-SF score at t1 and at t2 than participants in the CG with a small- to medium-sized effect (RESI = 0.26 [t1] & RESI = 0.26 [t2]). Regarding the NPI-NH, the adjusted GEE models showed no significant differences between the IG and CG at t1 and t2. Sensitivity analyses on individuals’ actual average weekly frequency of participating in the MAKS-mk + intervention (“as treated”) showed results comparable to the ITT analysis.

**Discussion:**

The results of the study show that the complex intervention had a positive effect on agitation and aggression in PlwDs or PlwMCIs with a small- to medium-sized effect. Overall, these findings should also contribute to improvement in the caregiving situation and living conditions of relatives and professional caregivers. As the intervention has demonstrated feasibility in the SHA setting, more consideration should be given to implementing it in everyday SHA care.

**Trial registration:**

ISRCTN89825211 (Registered prospectively, July 16, 2019).

**Supplementary Information:**

The online version contains supplementary material available at 10.1186/s12888-026-07810-x.

## Background

Along with a longer life expectancy, the number of care-dependent people is rising [1]. Due to societal changes (e.g., women in the workforce, an increasing number of people not living in a partnership or having children, greater local flexibility in living places, lower family cohesion), new kinds of care environments are needed in addition to caregiving at home (mostly by relatives) or nursing homes [2, 3].

Positive effects have been identified for care recipients and their well-being from care environments being as homelike as possible [4]. In Germany, one form of such a living arrangement is a *shared-housing arrangement* (SHA) [5, 6]. SHAs are defined as small and homelike care environments that consider residents’ needs and choices, offer person-centered care, and provide a daily routine that includes meaningful activities of daily living [5]. However, various international studies have found no clear overall advantage of SHAs in terms of a better care environment for people living with dementia (PlwDs) [7, 8].

Most of the SHAs have care-dependent PlwDs as a target group [9]. PlwDs live with cognitive symptoms, such as impairments of memory, orientation, and comprehension, and are commonly also affected by behavioral and psychological symptoms of dementia (BPSDs), such as agitation, aggression, anxiety, or depression [10]. SHAs can be a beneficial care environment for PlwDs: There is evidence of improved quality of life (QoL) in the long term in PlwDs, along with a reduction in BPSDs [7]. Nevertheless, adequate treatment of BPSDs in PlwDs is urgently needed because the presence of moderate or severe BPSDs – even after multivariate controlling – is significantly associated with an increased risk of mortality in PlwDs [11].

BPSDs challenge both PlwDs and their caregivers [12, 13] and are considered common causes for the hospitalization of PlwDs [14, 15]. The all-cause hospitalization risk is considered to be at least 1.42 times higher for PlwDs in comparison with people living without dementia in the same age group [14]. Hospitalization is highly problematic for PlwDs and often leads to negative effects, for example, declines in physical and cognitive functioning, but also an increased risk of falls, malnutrition, infections, delirium, or even death in the clinic [16–19]. At the same time, BPSDs often worsen in PlwDs during or after hospitalization [16, 20].

There has been only a small number of studies in SHAs, mostly with a descriptive research purpose [21, 22]. Intervention studies in this care setting are lacking. In general, there are a wide range of non-pharmacological interventions (with mixed results on their effects) for the treatment of BPSDs in PlwDs [23]. In other care settings, such as nursing homes or day care centers, non-pharmacological interventions have effectively reduced the symptom burden of BPSDs in PlwDs or PlwMCIs [24, 25].

Therefore, in the DemWG study presented here, we planned to implement an adapted version of the multimodal, psychosocial MAKS^®^ therapy – which has been shown to be effective for PlwDs in nursing homes and day cares [25–27] – in the setting of SHAs and to investigage whether its effects can be transferred to this setting. We aimed to reduce hospitalization as a primary outcome and to reduce BPSDs as a secondary outcome in PlwDs living in SHAs to optimize health care and improve QoL. Since the primary goal was to lower the frequency of hospitalization of PlwDs in SHAs, and two systematic reviews have shown that a single intervention (even if it was multimodal) would probably not be effective at lowering this risk [28, 29], we saw the need to embed an SHA-adapted version of the MAKS^®^ therapy into a complex intervention. Besides the non-pharmacological intervention for the PlwDs, we included two separate components that focused on the education of the healthcare professionals, i.e. nursing staff as well as general practitioners, providing healthcare in the SHAs – as recommended by the WHO [30] and as suggested by a recent meta-analysis on the efficacious treatment of BPSDs [31].

Results concerning the primary outcome have been published by Misonow, Wolf-Ostermann et al. [32]. Beyond that, results especially concerning QoL (QUALIDEM) and social participation will also published elsewhere (Misonow et al., in preparation). In this present work, we investigate the following research question: Is the complex intervention implemented in the DemWG study effective at lowering BPSDs (mainly agitation and aggression) of PlwDs and PlwMCIs in SHAs, i.e. the secondary outcome of the DemWG study?

## Methods

### Study design

The DemWG study was a prospective, multicenter, mixed-methods, cluster-randomized controlled trial (cRCT) with a waitlist control group design [33]. This paper focuses on the quantitative outcomes regarding the secondary outcome BPSDs. In addition to the study protocol [33], the study methodology has also been reported by Kratzer et al. [34], Misonow et al. [32], and Donath et al. [35]. The study had two study centers – one at the University of Bremen and one at the Friedrich-Alexander-Universität Erlangen-Nürnberg (FAU), both in Germany. The trial took place in 97 SHAs from 10 different German federal states from July 2020 to July 2022. The intervention period lasted 6 months. Due to restrictions from the ongoing pandemic of the SARS-CoV-2 virus, which varied from one SHA to another, participating SHAs randomized to the intervention group could determine a start date for the intervention between June 2020 and January 2021. Data were collected at baseline (t0), after the six-month intervention period (t1), and after an additional six-month follow-up period. All procedures were approved by the Ethics Committee of the University of Bremen (Ref. 2019-18-06-3) and were performed in accordance with national laws and the 2013 Declaration of Helsinki. In each participating SHA, on-site study coordinators (usually nursing staff) received 4 h of training on the study protocol, data collection, and screening process. This training enabled the coordinators to conduct the screening, collect the data, and act as a contact person between the study headquarters and the SHA. Participation in the study was voluntary, and participants and clusters were free to leave the study at any time without repercussions. Written informed consent was obtained from each participant, or from their legal guardian if applicable. The study was prospectively registered on July 16, 2019, in the ISRCTN registry (trial identification number ISRCTN89825211). For more information about the study design, please refer to the study protocol by Kratzer et al. [33].

### Recruitment and sample size

SHAs were recruited from July 2019 to October 2020 in all German states, though participating clusters were only located in Bavaria, Baden-Württemberg, Berlin, Brandenburg, Bremen, Mecklenburg-Vorpommern, Lower Saxony, Rhineland-Palatinate, and Schleswig-Holstein. SHAs and their outpatient care services were identified through their websites or entries in information systems and databases. Written information about the study was also sent to the ministries and administrative authorities of the different German federal states, nursing care centers (“Pflegestützpunkte”), and German Alzheimer’s societies, requesting that they forward the information to the SHAs. We interviewed all interested SHAs by telephone to include SHAs with at least three people living with dementia (PlwDs) or mild cognitive impairment (MCI) in the planned complex intervention and exclude SHAs with a focus on other mental disorders or intensive care before randomization (for the self-developed interview questions, please see Additional File 1). All interested SHAs that met the inclusion criteria signed a cooperation agreement. Recruitment ended in December 2020, and 97 SHAs and their 341 participants were included at baseline.

An a priori power analysis concerning the primary outcome of the DemWG study (hospital admissions) suggested including a minimum of 840 participants from 120 SHAs (60 in the intervention group and 60 in the control group, assuming seven participants per SHA). However, this study focused on the secondary outcome (i.e., BPSDs) rather than the primary outcome for which the power analysis was conducted (for additional details regarding the sample size estimation, see the study protocol [33]). The results of the trial concerning the primary outcome are published by Misonow, Wolf-Ostermann et al. [36].

### Eligibility of participants

All residents of each participating SHA were screened. The inclusion criteria were a psychometric verification of mild-to-moderate dementia (i.e., a Mini-Mental State Examination [MMSE] score of less than 24) or mild cognitive impairment (i.e., an MMSE score of greater than 23, but a Montreal Cognitive Assessment [MoCA] score of less than 24). Exclusion criteria included severe dementia (MMSE < 10), severe hearing or visual impairment, cognitive decline due to diseases other than dementia (e.g., schizophrenia, Korsakoff syndrome, or a history of severe major depression), permanent immobility, an inability to communicate verbally in German, and a history of more than one stroke.

### Randomization and blinding

The SHAs (clusters) were randomly assigned to the intervention (IG) or control (CG) group. Cluster randomization was chosen because the DemWG study’s non-pharmacological complex intervention addressed the SHAs and their nursing staff as a whole. Randomization was concealed and performed externally by the Competence Center of Clinical Trials of the University of Bremen (KKSB). The only information shared was the SHA code, the federal state in which the nursing home was located, and whether the SHA was rural or urban. The block sizes for the IG and CG were created randomly. The randomization list was created using R statistical software. Randomization was carried out per federal state and stratified by urban versus rural. The urban and rural strata were defined based on population figures. For this study, areas with fewer than 5,000 people were classified as rural and areas with more than 5,000 people were classified as urban. SHAs with a joint outpatient care service or joint general practitioners were assigned to the same cluster beyond this classification. After randomization, the KKSB transmitted the final group allocation to the study headquarters, which informed the participating SHAs of their allocation to the IG or CG. For organizational reasons, randomization was completed and communicated before screening.

Since we examined a non-pharmacological, complex intervention that addressed the entire SHA, neither the residents nor the trained professionals working in the SHAs could be blinded. However, the people who collected the data were not involved in implementing the MAKS-mk + psychosocial group intervention within the SHAs (component C of the complex intervention, which is described in the next section of this article).

### The complex intervention of the DemWG study

After the baseline assessment, each SHA in the intervention group (IG) received the DemWG study’s complex intervention. The SHAs in the control group (CG) continued to receive treatment as usual. For ethical reasons and due to the waitlist control group design, 12 months after the baseline assessment, SHAs in the CG were given the option to receive the complex intervention, including staff training, if they chose to. The complex intervention comprises three distinct and different components, which are described in the following section. For more information on the intervention and its development, see the study protocol by Kratzer et al. [33].

#### Component A – Education of people working in SHAs

Through the distribution of an informational brochure, Component A aimed to educate and raise awareness among SHAs regarding ambulatory care-sensitive conditions and health risk situations. Component A aimed to reduce hospital admissions, the primary outcome of the DemWG study, through education.

#### Component B – Continuing medical education (CME) of general practitioners

Component B involved the continuing medical education (CME) of general practitioners focusing on ambulatory care-sensitive conditions and preventing the hospitalization of PlwDs and PlwMCIs. The training was CME-certified by the Bavarian Medical Chamber (Bayerische Landesärztekammer) and published in the German peer-reviewed scientific journal Geriatrie-Report and is also available as a podcast. Component B aimed at reducing hospital admission, i.e. the primary outcome of the DemWG study, through an educational approach.

#### Component C – Multicomponent, psychosocial group intervention MAKS-MK+

Component C consisted of the multicomponent psychosocial group intervention MAKS-MK+. Designed for three to 12 participants, it consists of three modules: motor training (M), cognitive training (K), and fall prevention (+). The motor training module (M) included strength and muscle exercises for the upper limbs, such as ball and movement games derived from the original motor training module of the evidence-based MAKS^®^ therapy [26]. Cognitive training (K) included cognitive tasks such as memory exercises, Sudoku, picture puzzles, and quiz games. These tasks were projected digitally onto a large screen for the group to solve like a game, derived from the original cognitive training module of MAKS^®^ therapy [26]. Fall prevention (+) consisted of evidence-based exercises to increase muscle strength and balance but also coordination derived from the evidence-based OTAGO exercise program [37, 38]. The cognitive and physical group intervention was carried out in the shared living room of the SHA as a joint activity. According to a standardized manual, the MAKS-mk + intervention should be performed five days a week for six months. In the current intervention, each daily session began with 30 min of either fall prevention (Monday, Wednesday, and Friday) or motor training (Tuesday and Thursday), followed by a 30-minute session of cognitive training.

The SHAs in the intervention group received four hours of training on implementing the multicomponent psychosocial group intervention, MAKS-MK+, from a research associate at the study headquarters. The training was provided to at least two people in each SHA, usually nursing staff and occasionally volunteer assistants. The training included a presentation on the key elements of MAKS-mk + and role-playing exercises. The trainees also received detailed written instructions and manuals. All participating SHAs were provided with mini PCs to access the MAKS-mk + software, which they could use to present materials on a screen via projector or TV. They were also given financial compensation for implementing the MAKS-mk + intervention.

### Instruments

Trained nursing staff from the participating SHAs collected data using pseudonymized paper case report forms (CRFs). The CRFs were sent to the data monitoring committee at the KKSB, which checked the data for plausibility and completeness. The following instruments relevant to the present work were administered.

### Secondary outcomes of the DemWG study regarding BPSDs

#### Cohen-Mansfield Agitation Inventory – Short Form (CMAI-SF)

The German version of the CMAI-SF was used to measure agitation and aggression, which were the secondary outcomes of the DemWG study [34]. The CMAI-SF is a proxy-based instrument for assessing agitated and aggressive behaviors, derived from the original 29-item CMAI [39, 40]. Each item’s frequency was rated on a 5-point scale (total range from 14 to 70), with higher scores indicating more pronounced agitation and aggression. The CMAI is one of the most widely used instruments for assessing agitation in PlwDs [41]. Several studies have established the reliability and validity of the CMAI [39–43] and the CMAI-SF [34, 44, 45].

#### Neuropsychiatric Inventory – Nursing Home Version (NPI-NH)

Neuropsychiatric symptoms (BPSDs) as another secondary outcome of the DemWG study were measured with the German version of the NPI-NH [46, 47], which is derived from the Neuropsychiatric Inventory (NPI) [48]. The NPI is one of the most widely used instruments for assessing BPSDs [49, 50]. The NPI-NH is a proxy-based instrument for assessing the frequency (5-point scale ranging from 0 to 4) and severity (4-point scale ranging from 0 to 3) of 12 common BPSDs. A Frequency x Severity product score was built for each symptom. The total score was obtained by adding the Frequency x Severity scores of each item and ranged from 0 to 144 with higher scores indicating more pronounced BPSDs. The NPI-NH has been validated and is reliable [43, 46, 49, 51].

### Other measures (covariates)

The MMSE [52] and the MoCA [53] were used to screen for cognitive impairment. The MMSE is the most widely used cognitive screening test for dementia, and it has been shown to be reliable and valid [54–56]. The MoCA [53] is a widely used and validated screening tool for MCI [57, 58]. Scores on both instruments range from 0 to 30, with higher scores indicating better cognitive functioning.

Quality of life (QoL) as an important covariate was measured using the dementia-specific proxy-based QoL instrument QUALIDEM [59, 60]. QoL is assessed based on 37 items covering nine QoL dimensions. All items should be rated on a seven-point scale ranging from 0 (never) to 6 (very frequently) regarding observed behavior in the past week. The global score is calculated by adding the scores and transforming them into values ranging from 0 to 100 [61]. Higher scores indicate a better quality of life. The QUALIDEM has been shown to be reliable and valid [59, 60, 62–65].

Comorbidities were measured with the updated Charlson Comorbidity Index (CCI) developed by Quan et al. [66]. Twelve medical diagnoses were weighted according to their mortality-associated severity, resulting in a total score ranging from 0 to 24. Higher scores indicate an increased one-year mortality rate. The validity and reliability of the CCI have been confirmed [66–69].

Activities of daily living (ADLs) were measured using the German version of the Barthel Index [70, 71]. This instrument has 10 items and a total score ranging from 0 to 100. It is widely used, reliable, and valid for assessing ADL capabilities [70–72]. Higher scores indicate a better performance of ADL capabilities.

The following data were obtained from documentation provided by each SHA: sociodemographic data (e.g., age, sex); the average attendance rate of each participant in the MAKS-mk + intervention between the two measurement points (to quantify dose); the care level (Pflegegrad, ranging from 0 to 5; a higher level indicates greater impairment); the number of residents in each SHA; and the number of general practitioners and outpatient care services providing care at each SHA.

### Statistical analysis

Data analyses were performed using R software. Missing values due to loss to follow-up between t0 and t2 were imputed via iterative random forest imputation. At t1, 105 participants were lost to follow-up, so their scores were imputed using random forest imputation.

The generalized estimating equations (GEE) procedure was chosen for the primary statistical analysis. GEE models longitudinal or clustered data and can be used for categorical or continuous target variables. A major advantage of the GEE model over mixed models is that GEE calculations are usually simpler. Additionally, GEE model estimators are robust to losses of consistency in the event of an incorrect specification of the true underlying correlational structure of the data. Thus, their robustness gives them an additional advantage. In the regression models for the secondary outcomes (NPI-NH and CMAI-SF at t1), we computed a basic model, including the respective secondary outcome at t0, level of care at t0, and group affiliation (IG/CG) as fixed effects. Additionally, we computed an extended model that included all other independent variables at t0 that were significantly correlated (α < 0.10) with the respective secondary outcome at t1 in previously calculated bivariate correlational analyses, in addition to the variables in the basic model.

The primary data analytic strategy was intention-to-treat (ITT) as specified by the CONSORT statement [73, 74]. A sensitivity analysis was performed in which additional analyses were calculated using the as-treated sample. For each participant, the group allocation (IG vs. CG) was replaced by their actual average weekly frequency of participation (0 to 5 times per week) during the six-month intervention period.

The robust effect size index (RESI) by Vandekar et al. [75] was calculated as a measure of effect size because the RESI is generalizable across a wide range of models. Cohen’s effect size intervals — none-small (0.0–0.2), small–medium (0.2–0.5), and medium–large (0.5–0.8) — can be used to define similar regions for the RESI: small (0.0–0.1), small–medium (0.1–0.25), and medium–large (0.25–0.40).

A type I error rate (alpha) of less than 5% was considered statistically significant. However, since two main analyses were performed on one sample (i.e., NPI-NH and CMAI-SF), adjustments for multiple testing were necessary. We adjusted the p-values using the Benjamini-Hochberg method [76], which controls the false discovery rate more efficiently than the Bonferroni method.

## Results

### Description of clusters and study participants

A total of 97 SHAs and their associated 63 outpatient care services were included in the study at baseline (t0). The 54 SHAs in the IG had an average of 11.00 (*SD =* 6.80) employees working in the SHA and 9.54 (*SD =* 2.58) residents, whereas the 43 SHAs in the CG had an average of 11.50 (*SD =* 7.73) employees working in the SHA and 9.49 (*SD =* 1.85) residents. The intracluster correlation coefficient (ICC) can be considered low for the two secondary outcomes that were analyzed: CMAI-SF (ICC = 0.15) and NPI-NH (ICC = 0.15).

**Fig. 1:**
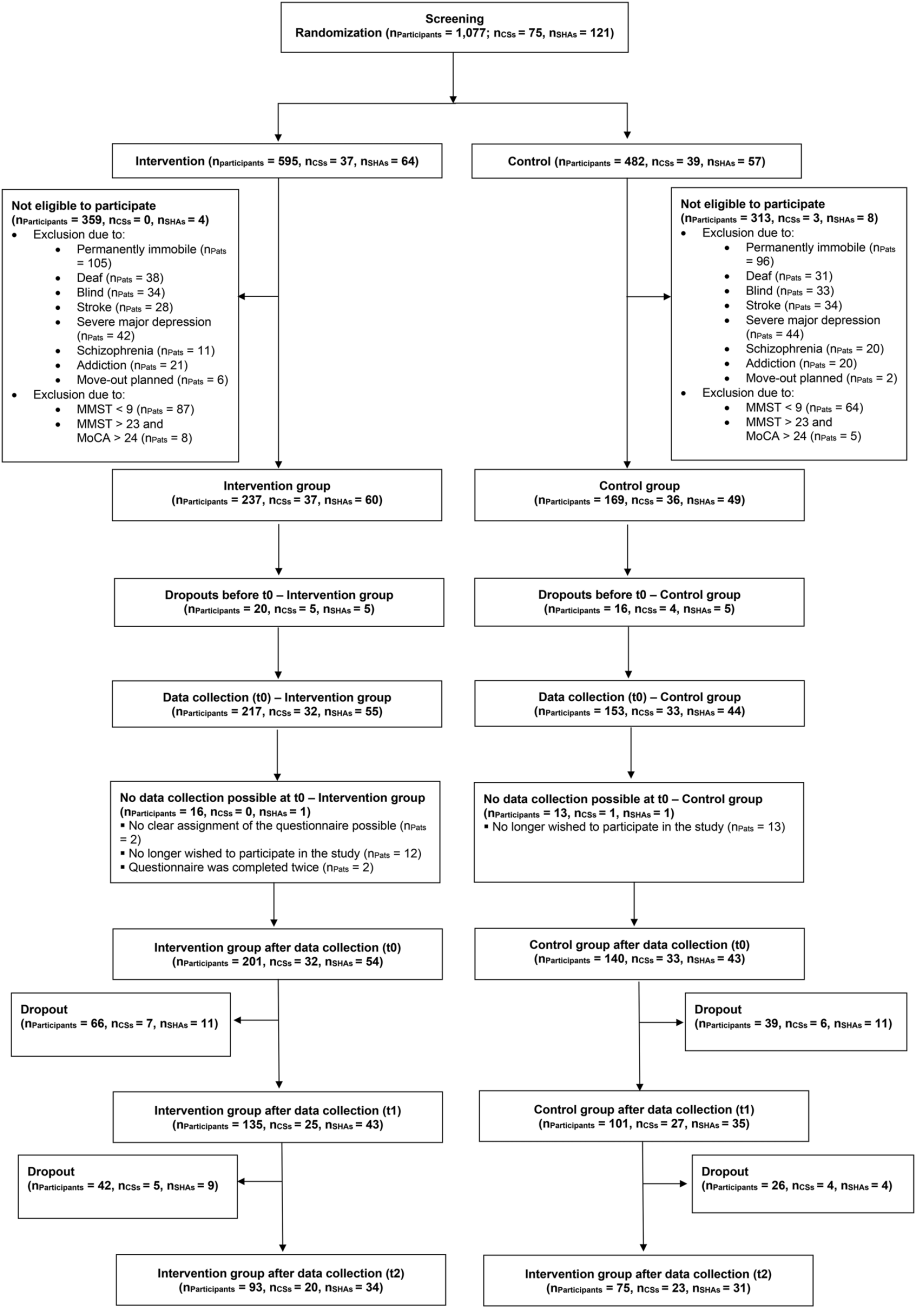
CONSORT flow chart of the DemWG Study. *Note.* PlwDs: People living with dementia; CS: outpatient care-service; SHA: shared-housing arrangement

However, 22 SHAs and 13 outpatient nursing care services dropped out between t0 and t1 because they no longer provided data in the data collection phase at t1 or no longer participated in the study (for details, see the CONSORT flow chart in Fig. 1).

The baseline characteristics of the study participants are shown in Table 1. The participants in the IG were 85 years old on average, and those in the CG were 84 years old. In both groups, the residents were predominantly female (IG: 80.6% and CG: 70.0%) and most of them belonged to care level 3 (IG: 48.8% and CG: 43.6%). The Charlson Comorbidity Index (CCI) was close to 3 for both the IG (3.3) and the CG (3.5) and therefore did not differ between the two groups. According to the Barthel Index, the participants in both groups could be classified as needing help on average (IG: 68 and CG: 62.9).

The severity of the participants’ cognitive impairment, assessed with the MMSE and MoCA, was also comparable between the IG and CG. At t0, participants were classified with MCI (*n* = 87, 25.5%, MMSE > 23 & MoCA < 24), mild dementia (*n* = 111, 32.6%, MMSE 23 − 18), moderate-severe dementia (*n* = 113, 33.1%, MMSE 17 − 10), or severe dementia (*n* = 30, 8.8%, MMSE < 10). The median time interval between the screening and the baseline data collection was 3 months (Range: 0 to 13 months) because the study had been interrupted by the outbreak of the COVID-19 pandemic in spring 2020. Therefore, although the degree of cognitive impairment at t0 corresponded to severe dementia for 30 individuals, these individuals had been classified as having moderate (*n* = 26) or mild dementia (*n* = 4) at screening and were therefore included in the study in accordance with the study protocol.

**Table 1:** Baseline characteristics of the study participants

Variable	Intervention group (*n* = 201)			Control group (*n* = 140)	
**Age**, *M* (*SD*)	85.40	(8.19)		83.8	(8.73)
**Sex**					
Female, *n* (%)	162	(80.6)		98	(70.0)
Male, *n* (%)	39	(19.4)		42	(30.0)
**Care level**, *Mdn* (*IQR*)	3.00	(1.00)		3.00	(1.00)
**MMSE sum score**, *M* (*SD*)	19.20	(6.02)		17.90	(6.81)
**Charlson Index**, *M (SD)*	3.27	(2.26)		3.51	(2.04)
**Barthel Index**, *M (SD)*	68.00	(24.90)		62.90	(26.90)
**Psychotropic drugs**					
Antidementia drugs (ATC code N06D), *n* (%)	61	(30.3)		41	(29.3)
Antidepressants (ATC code N06A), *n* (%)	89	(44.3)		67	(47.9)
Antipsychotics (ATC code N05A), *n* (%)	76	(37.8)		57	(40.7)
Tranquilizer & Hynpnotics/Sedatives (ATC codes N05B & N05C), *n* (%)	21	(10.4)		17	(12.1)

*Note. M* = Mean; *SD* = Standard deviation; Care level = higher scores indicate a higher need for care, Range: 0–5; MMSE = Mini-Mental State Examination, lower scores indicate more severe cognitive impairment, and a score between 0 and 9 indicates severe dementia, Range: 0–30; Charlson Index = Updated and validated Charlson Comorbidity Index by Quan et al., higher scores indicate a higher 1-year comorbidity-related mortality rate, Range: 0–24, whereby a score of 5 is associated with an 85% 1-year mortality risk; Barthel Index, Range: 0-100, higher scores indicate better performance of ADLs

As can be seen from Table 1, antidementia drugs (ATC code N06D), antidepressants (ATC code N06A), antipsychotics (ATC code N05A) as well as tranquilizers and hypnotics/sedatives (ATC codes N05B & N05C) were prescribed with comparable frequency in both groups.

Detailed information on the prevalence of neuropsychiatric symptoms, i.e. NPI-NH total scores and NPI-NH subscores as well as percentages of clinically relevant neuropsychiatric symptoms (NPI-NH score ≥ 4) in both groups are displayed in Additional File 2; detailed information on agitation and aggression symptoms, i.e. CMAI-SF total and subscores in both groups, are shown in Additional File 3. Participants in the control group were descriptively slightly more likely to have clinically relevant NPI-NH symptoms as well as at least one CMAI-SF symptom than participants in the intervention group.

The descriptive statistics for the CMAI-SF total score and the NPI-NH total score at t0, t1, and t2 can be found in Table 2.

**Table 2:** The secondary outcomes CMAI-SF and NPI-NH at t1, t1, and t2

Variable	Intervention (*n* = 201)		Control (*n* = 140)	
	M	SD	M	SD
**CMAI-SF**				
t0	18.27	6.12	19.91	7.55
t1	18.29	5.30	20.42	6.45
t2	17.75	4.31	20.99	9.56
**NPI-NH**				
t0	7.76	10.13	11.69	15.49
t1	8.72	11.01	11.01	11.79
t2	7.99	8.80	12.95	18.95

*Note. M* = Mean; *SD* = Standard deviation; CMAI-SF: Cohen-Mansfield Agitation Inventory – Short Form, Range: 14–70, higher scores indicate more pronounced agitation/aggression; NPI-NH: Neuropsychiatric Inventory – Nursing Home Version, Range: 0-144, higher scores indicate more pronounced BPSDs

Almost half (45%) of the participants in the IG took part in the MAKS-mk + intervention an average of five times per week, whereas 13.9% took part an average of four times per week; 13.9% an average of three times per week, 13.2% an average of two times per week, 11.9% an average of one time per week, and 2% never took part in MAKS-mk+. At t2, the most common frequencies of participation were still five times per week (27.7%), whereas 19.1% took part an average of two times per week, 17.1% an average of one time per week, 16.0% an average of three times per week, 13.8% an average of four times peer week, and 6.4% never took part in MAKS-mk+.

### Agitation and aggression – CMAI-SF

#### Descriptive statistics

The CMAI-SF total scores.

### ITT analysis

For the CMAI-SF total score at t1, no statistically significant difference was found between the CG and the IG in the basic model (*p* = .065). However, the extended model showed that participants from the IG had a significantly lower CMAI-SF total score at t1 than participants from the CG (see Table 3).

After adjusting for multiple testing by applying the Benjamini-Hochberg method, this effect remained significant (*p* = .011). The computed RESI of 0.26 can be considered a small- to medium-sized effect. A corresponding likelihood ratio test between the basic model and the extended model showed that the extended model was preferred.

For the CMAI-SF total score at t2, we found a statistically significant difference between the CG and IG in the basic model (Benjamini-Hochberg corrected *p* = .04) as well as in the extended model (Benjamini-Hochberg corrected *p* = .02). The computed RESI of 0.26 can be considered a small- to medium-sized effect.

### Sensitivity analysis – as treated

Considering the actual personal average weekly frequency of participation (0 to 5 times per week) in the 6-month intervention period, a significant effect of the intervention on the CMAI-SF total score at t1 could be observed for the basic model (*p* = .005; Benjamini-Hochberg corrected *p* = .010) as well as for the extended model (*p* = .002; Benjamini-Hochberg corrected *p* = .008). For the CMAI-SF total score at t2, no significant effect of the intervention could be found in both, the basic and the extended model.

**Table 3:** Extended, adjusted GEE model for the CMAI-SF score at t1 (ITT analysis)

	CMAI-SF total score (at t1)		
Predictors (at t0)	Estimates	CI	*p*
(Intercept)	11.05	3.11–19.00	**0.006**
CMAI-SF total score	0.43	0.15–0.72	**0.003**
Intervention group (reference: control group)	-1.95	-3.46 – -0.45	**0.011**
Care levels 1 & 2 (reference: care level 3)	-0.01	-1.36–1.35	0.991
Care levels 4 & 5 (reference: care level 3)	-0.82	-2.10–0.46	0.210
Year of birth	0.04	-0.03–0.11	0.271
Number of care services in the SHA	-16.96	-38.83–4.90	0.128
Number of general practitioners for the SHA	1.62	-3.26–6.49	0.515
Number of residents in the SHA	1.17	-0.43–2.77	0.151
QUALIDEM total core	0.88	-4.93–6.68	0.767
MMSE total score	-0.15	-0.25 – -0.06	**0.002**
NPI-NH total score	-0.04	-0.13–0.05	0.404
Number of antidementia drugs	0.66	-0.84–2.15	0.388
Number of antipsychotics	1.44	0.23–2.65	**0.020**

Note. CMAI-SF: Cohen-Mansfield Agitation Inventory – Short Form, Range: 14–70, higher scores indicate more pronounced agitation/aggression; Care level was included in the model as a categorical variable, with care level 3 as the reference category; SHA: Shared-housing arrangement; Number of general practitioners for the SHA: Number of general practitioners who are responsible for the different residents of the relevant SHA; QUALIDEM: dementia-specific quality of life (QoL) instrument, Range: 0-100, higher scores indicate better QoL; MMSE: Mini-Mental State Examination, Range: 0–30, lower scores indicate higher cognitive impairment; NPI-NH: Neuropsychiatric Inventory – Nursing Home Version, Range: 0-144, higher scores indicate more pronounced BPSDs

### BPSDs – NPI-NH

#### ITT analysis

For the NPI-NH total score at t1, no statistically significant difference was found between the CG and the IG in the basic model (*p* = .120) or in the extended model (*p* = .123). The adjusted GEE model for the extended model in the ITT sample is presented in Table 4. For the NPI-NH total score at t2, a significant effect of the intervention was only detected in the basic model (Benjamini-Hochberg corrected *p* = .049), but not in the extended, adjusted model (*p* = .110).

**Table 4:** Extended, adjusted GEE model for the NPI-NH total score at t1 (ITT analysis)

	NPI-NH total score (at t1)		
Predictors (at t0)	Estimates	95% CI	*p*
(Intercept)	22.36	1.37–43.36	**0.037**
NPI-NH total score	0.25	-0.00–0.50	0.054
Intervention group (reference: control group)	-2.17	-4.82–0.47	0.107
Care levels 1 & 2 (reference: care level 3)	-0.78	-3.72–2.16	0.602
Care levels 4 & 5 (reference: care level 3)	1.36	-1.25–3.97	0.306
Number of care services in the SHA	-9.05	-49.41–31.31	0.660
Number of residents in the SHA	-0.06	-14.06–13.95	0.994
QUALIDEM total score	-20.93	-35.07 – -6.80	**0.004**
CMAI-SF total score	0.07	-0.34–0.48	0.736
Number of antipsychotics	2.83	0.68–4.99	**0.010**

Note. NPI-NH: Neuropsychiatric Inventory – Nursing Home Version, Range: 0-144, higher scores indicate more pronounced behavioral and psychological symptoms in dementia; SHA: Shared-housing arrangement; Charlson Index = Updated and validated Charlson Comorbidity Index by Quan et al., higher scores indicate a higher 1-year comorbidity-related mortality rate, Range: 0–24, whereby a score of 5 is associated with an 85% 1-year mortality risk; QUALIDEM: dementia-specific quality of life (QoL) instrument, Range: 0-100, higher scores indicate better QoL; CMAI-SF: Cohen-Mansfield Agitation Inventory – Short Form, Range: 14–70, higher scores indicate more pronounced agitation/aggression

### Sensitivity analysis – as treated

Considering the actual personal average weekly frequency of participation (0 to 5 times per week) in the 6-month intervention period, a significant effect of the intervention on the NPI-NH total score at t1 was observed only in the basic model (Benjamini-Hochberg corrected *p* = .011), but not in the extended model (*p* = .071).

## Discussion

Our complex intervention included both care-centered components delivered to the professional caregivers in the SHAs and the associated general practitioners, as well as individual non-pharmacological components delivered to PlwDs and PlwMCIs in the SHAs. We found that this complex intervention had a small- to medium-sized effect (in terms of the RESI [75]) on agitation and aggression as assessed with the CMAI-SF. The size of the effect corresponds to a Cohen’s *d* value of 0.52 under the assumptions of equal sample proportions and homoscedasticity of the model [75]. This value is far above the range that would be expected for similar interventions in the literature – even though previous intervention studies have not investigated the SHA setting, and most studies did not publish effect sizes: A complex intervention in Australian nursing homes including training based on person-centered care for the staff combined with an individual non-pharmacological component for the residents also showed a significant effect in the CMAI, although no effect size was reported [77]. Similarly, a randomized controlled trial (RCT) in Turkey with informal at-home caregivers who were trained to provide person-centered care and to deliver tailored stimulating interventions for PlwDs reported significant effects for the outcome measures CMAI and NPI in the sense of a reduction in symptoms; the size of the effect was not published [78]. A Cochrane review in 2018 [79] on tailored interventions in long-term care facilities, which are “likely to be complex interventions,” concluded that small effects (SMD = 0.21) can be expected for the outcome “challenging behavior” in PlwDs and included eight studies, seven of which were RCTs. Furthermore, a systematic review of systematic reviews on non-pharmacological interventions for PlwDs concerning the outcome BPSDs showed some evidence of a reduction in BPSDs for multicomponent interventions when the interventions were conceptualized as a multidisciplinary approach [23]. An earlier systematic review of the effect of integrated interventions with multiprofessional care concepts focusing on behavioral problems included eight studies, half of them RCTs, and identified beneficial effects in seven of eight trials [80].

We did not observe a significant change in the NPI-NH score from the complex intervention in the adjusted statical models – despite the observed effects in the unadjusted GEE models. In our view, there are two possible explanations for this lack of significance. First, the frequency of clinically relevant symptoms assessed with the NPI-NH (i.e., NPI-NH Frequency x Severity score > 3 [81]) was rather low in our sample, ranging from 2.7% of participants with clinically relevant hallucinations up to 12.0% of participants with clinically relevant appetite/eating changes. Thus, the symptom load might not have been high enough for a change to be detectable – in that there may have been a floor effect. Second, we might interpret the finding to mean that the intervention affected agitation and aggression (assessed with the CMAI-SF) but that the BPSD symptoms and behaviors measured by the NPI-NH are much broader. Thus, it is possible that not all of the symptoms we investigated with the NPI-NH were affected by the intervention. The literature also reported that it is possible for a non-pharmacological intervention involving physical exercises – as was at least partly the case in our intervention – to fail to lead to a measurable effect on NPI in PlwDs [82]. In addition, the sample consisted of 70% to 80% women. Given the fact that among PlwDs, the prevalence of the BPSDs is higher for men than for women [83, 84], it is conceivable that the intervention would produce greater effects on BPSDs in general in a more balanced sample.

### Strengths

The DemWG study was the first cRCT to investigate the effects of a complex intervention on BPSDs in the setting of SHAs. Agitation and aggression were assessed with a validated instrument [34] that is widely used (gold-standard) [34, 44, 45]. Thus, results could be included in future meta-analyses. The study, a cRCT, was planned and implemented to minimize biases as determined by the Cochrane Risk of Bias evaluation. However, a cRCT with a complex, non-pharmacological intervention cannot be blinded for the participants. As suggested by Lorenz et al. [85] for cRCTs, at least the assessment of the outcomes was not carried out by the study staff or the therapists who conducted the MAKS-mk + intervention. In addition, cRCTs suffer from the disadvantage of reduced sample size and statistical power in comparison with individually randomized trials [85]. However, in this study, there were a large number of clusters (97 SHAs) randomized with a small number of individuals in each (*M* = 3.51), thus leading to a higher statistical power than fewer clusters with many individuals would have provided [86]. Statistical analyses were carried out to the highest established standards [87] with the ITT sample and with models that consider the multilevel structure of the data. Furthermore, we used a robust estimate of the effect size corresponding to the models that were analyzed, we corrected the family-wise error rate because we tested more than one outcome, we imputed missing values in order to minimize bias, and we calculated sensitivity analyses. The study was registered prospectively (before first-patient-in), and the study protocol was published shortly after the study began.

### Limitations

First, the study is not representative of all PlwDs or PlwMCIs in SHAs in Germany, as the SHAs could not be randomly selected from the total number of SHAs because there is no compulsory registration for SHAs in Germany. In addition, BPSDs were only a secondary outcome in the DemWG study, and therefore, several aspects of the study (e.g., sample size estimation) were not centered around the outcome of BPSDs but were instead focused on the primary outcome. The communication of which group (IG or CG) the SHAs were allocated to took place before the staff that carried out the screening and assessment had been trained. Thus, a possible recruitment bias (subsampling bias) cannot be ruled out. A substantial number of individuals dropped out of the study at t1 and t2 (especially in the years 2020 and 2021) due to death and other factors that were likely associated with the COVID-19 pandemic. To consider this attrition, we carried out both a complete case analysis and an ITT analysis with imputed data, and we computed bivariate preanalyses to check for significant associations with the outcomes and thus identified control variables that needed to be included in the final multivariate models. Furthermore, also due to the pandemic and its restrictions, the MAKS-mk + intervention (i.e., component C of the complex intervention) was not implemented in every SHA in the desired dose (five times per week). We took this fact into account by computing sensitivity analyses with the dose variable, that is, the actual personal average weekly frequency of participation (0 to 5 times per week) in the 6-month intervention period. Unfortunately, there is no data available for the education of nursing staff (component A) and general practitioners (component B). Thus, it remains unclear to which extent these components had an effect on BPSD.

## Conclusions

The results of the present DemWG study show that the complex intervention, including MAKS-mk+, which was simultaneously targeted toward residents of the SHAs and toward the nursing staff, was able to positively affect the agitation and aggression of PlwDs or PlwMCIs with a small- to medium-sized effect. Overall, this finding should contribute to an improvement in the care situation and living conditions of those affected, their relatives, and their caregivers. As the intervention has been shown to be feasible in the SHA setting, more consideration should be given to implementing it in everyday SHA care.

## Supplementary Information

Below is the link to the electronic supplementary material.

Supplementary Material 1

Supplementary Material 2

Supplementary Material 3

## Data Availability

The data sets that were generated or analyzed for the current study will be available upon request from Stephan Kloep (kloep@uni-bremen.de). Data will be available within the time interval of 12 months until 36 months after the article is published. The data will be provided for noncommercial research purposes only to researchers with a proposal that was peer-reviewed and approved by an independent review committee. The inquiring researchers have to present an analysis plan and state the research purpose for which the data are needed (e.g., meta-analysis). Data will be available through the data warehouse of the University of Bremen without any additional investigator support. The data that can be provided refer solely to the data underlying the results presented in this manuscript. They will be completely anonymized, linkage to the stored data with personal information will not be possible. Therefore, case-specific additional information/clarification can no longer be provided.

## Abbreviations

ADLs: Activities of Daily Living
BPSDs: Behavioral and Psychological Symptoms of Dementia
CCI: Charlson Comorbidity Index
CG: Control Group
CMAI: Cohen-Mansfield Agitation Inventory
CMAI-SF: Cohen-Mansfield Agitation Inventory-Short Form
CME: Continuing Medical Education
GEE: Generalized Estimating Equations
IG: Intervention Group
ITT: Intention-to-treat
KKSB: Competence Center of Clinical Trials of the University of Bremen
MCI: Mild Cognitive Impairment
MMSE: Mini-Mental State Examination
MoCA: Montreal Cognitive Assessment
NPI: Neuropsychiatric Inventory
NPI-NH: Neuropsychiatric Inventory-Nursing Home Version
PlwDs: People living with Dementia
PlwMCIs: People living with Mild Cognitive Impairment
QoL: Quality of Life
cRCT: Cluster-Randomized Controlled Trial
RESI: Robust Effect Size Index
SHAs: Shared-Housing Arrangements
WHO: World Health Organization
