# Supplementary material for: Effects of a complex intervention on agitation and aggression in people living with dementia and mild cognitive impairment in shared-housing arrangements: results for a secondary outcome of the multicenter, cluster-randomized controlled DemWG study
Source: BMC Psychiatry. 2026 Jan 24;26:88. doi: 10.1186/s12888-026-07810-x (PMC12849605; doi:10.1186/s12888-026-07810-x)
Supplement: Supplementary file 1 — Supplementary Material 1 [file 12888_2026_7810_MOESM1_ESM.docx]

**Additional File 1**

**Interview guideline for the recruitment interviews with interested shared-housing arrangements**

*How many people with dementia or mild cognitive disorder live in your SHA?*

*Are you an SHA specialized on intensive care or on mental disorders other than chronic progredient cognitive diseases (like dementia)?*
