# Supplementary material for: Effects of a complex intervention on agitation and aggression in people living with dementia and mild cognitive impairment in shared-housing arrangements: results for a secondary outcome of the multicenter, cluster-randomized controlled DemWG study
Source: BMC Psychiatry. 2026 Jan 24;26:88. doi: 10.1186/s12888-026-07810-x (PMC12849605; doi:10.1186/s12888-026-07810-x)
Supplement: Supplementary file 2 — Supplementary Material 2 [file 12888_2026_7810_MOESM2_ESM.docx]

**Additional File 3**

**Table AF2: Prevalence of agitation/aggression in CMAI-SF in the sample of the DemWG-study (N = 341)**

| CMAI-SF Items* | CMAI-SF  Item score: M (SD)  Total sample | CMAI-SF  Score > 1:  n (%)  Total sample | CMAI-SF  Item score: M (SD)  IG (n = 201) | CMAI-SF  Score > 1:  n (%)  IG (n = 201) | CMAI-SF  Item score: M (SD)  CG (n = 140) | CMAI-SF  Score > 1:  n (%)  CG (n = 140) |
| --- | --- | --- | --- | --- | --- | --- |
| Item 1: cursing/verbal aggression | 1.64 (1.00) | 122 (35.8) | 1.50 (0.91) | 58 (28.9) | 1.83 (1.09) | 64 (45.7) |
| Item 2: hitting, kicking, pushing, biting, scratching, aggressive spitting | 1.14 (0.50) | 29 (8.5) | 1.08 (0.41) | 9 (4.5) | 1.21 (0.61) | 20 (14.3) |
| Item 3: grabbing onto people, throwing things, tearing things, or destroying property | 1.16 (0.58) | 29 (8.5) | 1.11 (0.47) | 14 (7.0) | 1.22 (0.71) | 15 (10.7) |
| Item 4: other aggressive behaviours or self abuse including: intentional falling, making verbal or physical sexual advances, eating/ drinking/ chewing inappropriate substances, hurt self or other | 1.07 (0.32) | 19 (5.6) | 1.03 (0.18) | 7 (3.5) | 1.11 (0.45) | 12 (8.6) |
| Item 5: pace, aimless wandering, trying to get to a different place (e.g. out of the room/building) | 1.49 (1.08) | 75 (22.0) | 1.49 (1.08) | 43 (21.4) | 1.49 (1.09) | 32 (22.9) |
| Item 6: general restlessness, performing repetitious mannerisms, tapping, strange movements | 1.34 (0.90) | 56 (16.4) | 1.28 (0.84) | 25 (12.4) | 1.44 (0.97) | 31 (22.1) |
| Item 7: inappropriate dress or disrobing | 1.35 (0.89) | 56 (16.4) | 1.35 (0.90) | 32 (15.9) | 1.34 (0.87) | 24 (17.1) |
| Item 8: handling things inappropriately | 1.31 (0.75) | 61 (17.9) | 1.28 (0.73) | 32 (15.9) | 1.34 (0.77) | 29 (20.7) |
| Item 9: constant request for attention or help | 1.70 (1.17) | 113 (33.1) | 1.62 (1.12) | 61 (30.3) | 1.81 (1.24) | 52 (37.1) |
| Item 10: repetitive sentences, calls, questions, or words | 1.52 (1.09) | 75 (22.0) | 1.45 (1.06) | 35 (17.4) | 1.63 (1.12) | 40 (28.6) |
| Item 11: complaining, negativism, refusal to follow directions | 1.52 (0.95) | 104 (30.5) | 1.43 (0.85) | 53 (26.4) | 1.66 (1.07) | 51 (36.4) |
| Item 12: strange noises (weird laughter or crying) | 1.09 (0.42) | 18 (5.3) | 1.06 (0.33) | 8 (4.0) | 1.12 (0.52) | 10 (7.1) |
| Item 13: hiding things, hoarding things | 1.45 (0.93) | 75 (22.0) | 1.43 (0.91) | 43 (21.4) | 1.46 (0.96) | 32 (22.9) |
| Item 14: screaming | 1.18 (0.64) | 32 (9.4) | 1.14 (0.60) | 13 (6.5) | 1.24 (0.70) | 19 (13.6) |
| Total score CMAI-SF | 18.95 (6.78) | 231 (67.7) | 18.27 (6.12) | 128 (63.7) | 19.91 (7.55) | 103 (73.6) |

*Note.* *each item is rated on a 5-point scale (ranging from 1 “never” to 5 “several times daily or more”), total score can possibly range from 14 to 70, higher scores indicating more pronounced agitation; CMAI-SF Score > 1 = persons showed this behavior at least “less than once a week”.

CMAI-SF: Cohen-Mansfield Agitation Inventory – Short Form; IG: intervention group; CG: control group; M: arithmetic mean; SD: standard deviation
