# Supplementary material for: Effects of a complex intervention on agitation and aggression in people living with dementia and mild cognitive impairment in shared-housing arrangements: results for a secondary outcome of the multicenter, cluster-randomized controlled DemWG study
Source: BMC Psychiatry. 2026 Jan 24;26:88. doi: 10.1186/s12888-026-07810-x (PMC12849605; doi:10.1186/s12888-026-07810-x)
Supplement: Supplementary file 3 — Supplementary Material 3 [file 12888_2026_7810_MOESM3_ESM.docx]

**Additional File 2**

**Table AF1: Prevalence of neuropsychiatric symptoms in NPI-NH in the sample of the DemWG-study (N = 341)**

| NPI-NH Subscales | NPI-NH  Item score: M (SD)  Total sample | NPI-NH  Score > 0:  n (%)  Total sample | NPI-NH  Score ≥ 4:  n (%)  Total sample | NPI-NH  Item score: M (SD)  IG (n = 201) | NPI-NH  Score > 0:  n (%)  IG (n = 201) | NPI-NH  Score ≥ 4:  n (%)  IG (n = 201) | NPI-NH  Item score: M (SD)  CG (n = 140) | NPI-NH  Score > 0:  n (%)  CG (n = 140) | NPI-NH  Score ≥ 4:  n (%)  CG (n = 140) |
| --- | --- | --- | --- | --- | --- | --- | --- | --- | --- |
| Delusions | .54 (1.68) | 51 (15.0) | 21 (6.2) | .37 (1.27) | 24 (11.9) | 8 (4.0) | .78 (2.12) | 27 (19.3) | 13 (9.2) |
| Hallucinations | .25 (1.15) | 24 (7.0) | 9 (2.7) | .15 (0.83) | 8 (4.0) | 4 (2.0) | .40 (1.48) | 16 (11.4) | 5 (3.5) |
| Aggression | 1.00 (2.30) | 85 (24.9) | 38 (11.2) | .76 (1.88) | 41 (20.4) | 17 (8.5) | 1.34 (2.76) | 44 (31.4) | 21 (15.0) |
| Depression | 1.18 (2.14) | 139 (40.8) | 33 (9.8) | 1.09 (2.09) | 78 (38.8) | 16 (8.0) | 1.31 (2.21) | 61 (43.6) | 17 (12.1) |
| Anxiety | .70 (1.79) | 75 (22.0) | 20 (5.9) | .53 (1.36) | 40 (19.9) | 8 (4.0) | .93 (2.26) | 35 (25.0) | 12 (8.5) |
| Euphoria | .28 (1.24) | 26 (7.6) | 11 (3.3) | .24 (1.17) | 13 (6.5) | 5 (2.5) | .34 (1.34) | 13 (9.3) | 6 (4.2) |
| Apathy | .90 (2.22) | 79 (23.2) | 28 (8.3) | .62 (1.64) | 40 (19.9) | 10 (5.0) | 1.31 (2.80) | 39 (27.9) | 18 (12.8) |
| Disinhibition | .61 (1.83) | 57 (16.7) | 23 (6.8) | .50 (1.47) | 33 (16.4) | 11 (5.5) | .76 (2.25) | 24 (17.1) | 12 (8.5) |
| Irritability | 1.29 (2.29) | 132 (38.7) | 39 (11.5) | .99 (1.85) | 69 (34.3) | 15 (7.5) | 1.72 (2.75) | 63 (45.0) | 24 (17.1) |
| Aberrant motor behavior | .85 (2.56) | 47 (13.8) | 30 (8.8) | .67 (2.27) | 22 (10.9) | 14 (7.0) | 1.11 (2.91) | 25 (17.9) | 16 (11.4) |
| Nighttime behavior | .83 (1.99) | 77 (22.6) | 27 (7.9) | .79 (1.88) | 46 (22.9) | 14 (7.0) | .89 (2.15) | 31 (22.1) | 13 (9.3) |
| Appetite and eating changes | .95 (2.43) | 67 (19.6) | 41 (12.1) | 1.05 (2.55) | 43 (21.4) | 25 (12.5) | .81 (2.24) | 24 (17.1) | 16 (11.4) |
| Total score NPI-NH | 9.37 (12.74) | 262 (76.8) | 193 (56.6) | 7.76 (10.13) | 149 (74.1) | 105 (52.1) | 11.69 (15.49) | 113 (80.6) | 88 (62.8) |

*Note.* NPI-NH: Neuropsychiatric Inventory – Nursing Home Edition; NPI-NH Score ≥ 4: clinically relevant symptoms; IG: intervention group; CG: control group; M: arithmetic mean; SD: standard deviation
